# Monitoring COVID-19 on Social Media: Development of an End-to-End Natural Language Processing Pipeline Using a Novel Triage and Diagnosis Approach

**DOI:** 10.2196/30397

**Published:** 2022-02-28

**Authors:** Abul Hasan, Mark Levene, David Weston, Renate Fromson, Nicolas Koslover, Tamara Levene

**Affiliations:** 1 Department of Computer Science and Information Systems Birkbeck, University of London London United Kingdom; 2 Barnet General Hospital London United Kingdom

**Keywords:** COVID-19, conditional random fields, disease detection and surveillance, medical social media, natural language processing, severity and prevalence, support vector machines, triage and diagnosis

## Abstract

**Background:**

The COVID-19 pandemic has created a pressing need for integrating information from disparate sources in order to assist decision makers. Social media is important in this respect; however, to make sense of the textual information it provides and be able to automate the processing of large amounts of data, natural language processing methods are needed. Social media posts are often noisy, yet they may provide valuable insights regarding the severity and prevalence of the disease in the population. Here, we adopt a triage and diagnosis approach to analyzing social media posts using machine learning techniques for the purpose of disease detection and surveillance. We thus obtain useful prevalence and incidence statistics to identify disease symptoms and their severities, motivated by public health concerns.

**Objective:**

This study aims to develop an end-to-end natural language processing pipeline for triage and diagnosis of COVID-19 from patient-authored social media posts in order to provide researchers and public health practitioners with additional information on the symptoms, severity, and prevalence of the disease rather than to provide an actionable decision at the individual level.

**Methods:**

The text processing pipeline first extracted COVID-19 symptoms and related concepts, such as severity, duration, negations, and body parts, from patients’ posts using conditional random fields. An unsupervised rule-based algorithm was then applied to establish relations between concepts in the next step of the pipeline. The extracted concepts and relations were subsequently used to construct 2 different vector representations of each post. These vectors were separately applied to build support vector machine learning models to triage patients into 3 categories and diagnose them for COVID-19.

**Results:**

We reported macro- and microaveraged F_1_ scores in the range of 71%-96% and 61%-87%, respectively, for the triage and diagnosis of COVID-19 when the models were trained on human-labeled data. Our experimental results indicated that similar performance can be achieved when the models are trained using predicted labels from concept extraction and rule-based classifiers, thus yielding end-to-end machine learning. In addition, we highlighted important features uncovered by our diagnostic machine learning models and compared them with the most frequent symptoms revealed in another COVID-19 data set. In particular, we found that the most important features are not always the most frequent ones.

**Conclusions:**

Our preliminary results show that it is possible to automatically triage and diagnose patients for COVID-19 from social media natural language narratives, using a machine learning pipeline in order to provide information on the severity and prevalence of the disease for use within health surveillance systems.

## Introduction

### Overview

During the ongoing coronavirus pandemic, hospitals have been continuously at risk of being overwhelmed by the number of people developing serious illness. People in the United Kingdom were advised to stay at home if they had coronavirus symptoms and to seek assistance through the National Health Service (NHS) helpline if they needed to [1]. Consequently, there is an urgent need to develop novel, practical approaches to assist medical staff. A variety of methods have been recently developed that involve *natural language processing* (NLP) techniques; the concerns of these methods range from the level of the individual (see, for example, [2,3]) up to the population level [4,5].

Herein, we take a diagnostic approach and propose an end-to-end NLP pipeline to automatically triage and diagnose COVID-19 cases from patient-authored medical social media posts. The triage may inform decision makers about the severity of COVID-19, and diagnosis could help in gauging the prevalence of infections in the population. Attempting a clinical diagnosis of influenza, or in our case a diagnosis of COVID-19, purely based on the information provided in a social media post is unlikely to be sufficiently accurate to be actionable at an individual level, since the quality of this information will be typically noisy and incomplete. However, it is not necessary to have actionable diagnoses at the individual level in order to identify interesting patterns at the population level, which may be useful within public health surveillance systems. For example, text messages from the microblogging site Twitter were used to identify influenza outbreaks [6]. In addition, Twitter data in conjunction with a US Centers for Disease Control and Prevention (CDC) data set were used to predict the percentage of influenza-like illness in the US population [7].

One of our key concerns is in the production of a high-quality human-labeled data set on which to build our pipeline. Here, we give a brief overview of our pipeline and how we developed our data set. The first step in the pipeline was attained by developing an annotation application that detects and highlights COVID-19-related symptoms with their severity and duration in a social media post, henceforth collectively termed as *concepts*. During the second step, relations between symptoms and other relevant concepts were also automatically identified and annotated. For example, *breathing hurts* is a symptom, which is related to a body part, the *upper chest area*.

**Figure 1:**
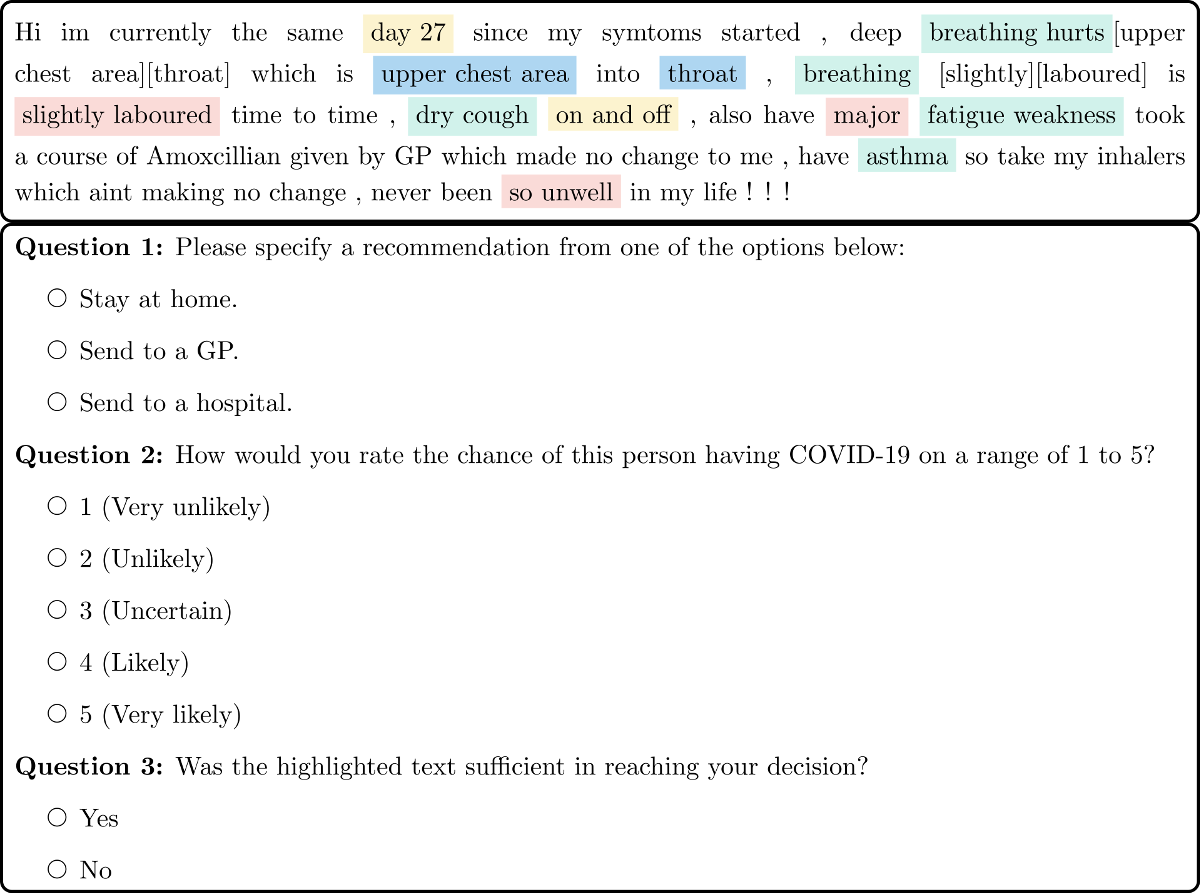
A patient-authored social media post is annotated with symptoms (light green), affected body parts (pale blue), duration (light yellow), and severities (pink). The phrases in square brackets show relations between a symptom and a body part/duration/severity when the distance is greater than 1. This annotated post was presented to 3 doctors to triage and diagnose the author of the post by answering questions 1 and 2, respectively. GP: general physician.

One author manually annotated our data with concepts and relations, allowing us to present posts highlighted with identified concepts and relations to 3 experts, along with several questions, as shown in Figure 1. The first question asked the experts to triage a patient into 1 of the following 3 categories: *Stay at home, Send to a GP* (where GP stands for general physician), or *Send to a hospital*. The second question asked to diagnose the likelihood of COVID-19 on a Likert scale of 1-5 [8].

The 3 experts are junior doctors working in the United Kingdom who were redeployed to work on COVID-19 wards during the first wave of the pandemic, between March and July 2020. Their roles involved the diagnosis and management of patients with COVID-19, including patients who were particularly unwell and required either noninvasive or invasive ventilation. There were some training sessions organized for doctors working in COVID-19 wards. However, these were only provided toward the end of the first wave, as there was initially little knowledge of the virus and how to treat it. In the hospital, the doctors followed local protocols, which were adjusted as more experience was gained about the virus.

We also asked the doctors to indicate whether the highlighted text presented is sufficient in reaching their decision in order to understand its usefulness when we incorporate it in the annotation interface. The annotations were found to be sufficient in as many as 85% of the posts, on average, as indicated by the doctors’ answers to question 3 in Figure 1.

The posts labeled by the doctors were then used to construct 2 types of predictive machine learning model using *support vector machines* (SVMs) [9,10]; see the Step 4: Triage and Diagnosis subsection in the Methods section. The *triage models* use hierarchical binary classifiers, which consider the risk averseness or tolerance of the doctors when making the diagnosis [11]. The *diagnostic models* first calculate the probability of a patient having COVID-19 from doctors’ ratings. The probabilities are then used to construct 3 different decision functions for classifying *COVID* and *NO_COVID* classes; these are detailed in the Problem Setting subsection in the Methods section.

We trained the SVM models in 2 different ways: first with ground-truth annotations and second using predictions from the concept and relation extraction step described before. Predictions obtained from the concept extraction step make use of *conditional random fields* (CRFs) [12]; see the Step 1: Concept Extraction subsection in the Methods section for implementation details. Relations are obtained from these predicted concepts using an unsupervised *rule-based* (RB) classifier [13]; see the Step 2: Relation Extraction subsection in the Methods section.

We also discussed the feature importance obtained from the constructed COVID-19 diagnostic models and compared it with the most frequent symptoms from Sarker et al [4] and our data set. We found that symptoms such as anosmia/ageusia (loss of smell/taste) rank in the top 5 most important features, whereas they do not rank in the top 5 most frequent symptoms; see the Discussion section. Overall, we made several contributions as follows:

- We showed that it is possible to take an approach that aims at disease detection to augment public health surveillance systems, by constructing machine learning models to triage and diagnose COVID-19 from patients' natural language narratives. To the best of our knowledge, no other previous work has attempted to triage or diagnose COVID-19 from social media posts.
- We also built an end-to-end NLP pipeline by making use of automated concept and relation extraction. Our experiments showed that the models built using predictions from concept and relation extraction produce similar results to those built using ground-truth human concept annotation.

### Related Work

Data derived from social media have been successfully used to facilitate the detection of influenza epidemics [6,7]. In addition, Edo-Osagie et al [14] provide a thorough review of the use of Twitter in public health surveillance for the purpose of monitoring, detecting, and forecasting influenza-like illnesses. Since the start of the COVID-19 pandemic, a number of mobile application–based, self-reported symptom tools have emerged to track novel symptoms [15]. The mobile application in Menni et al [16] applied logistic regression (LR) to predict the percentage of probable infected cases among the total application users in the United States and United Kingdom combined. Mizrahi et al [17] performed a statistical analysis on primary care electronic health record (EHR) data to find longitudinal dynamics of symptoms prior to and throughout the infection.

At an individual diagnostic level, Zimmerman et al [18] applied classification and regression trees to determine the likelihood of symptom severity of influenza in clinical settings. Moreover, machine learning algorithms, such as decision trees, have shown promising results in detecting COVID-19 from blood test analyses [19]. Here, we focus on features extracted from a textual source to triage and diagnose COVID-19 for the purpose of providing population-level statistics in the context of public health surveillance. Studies related to our work deploy features obtained from online portals, telehealth visits, and structured and unstructured patient/doctor notes from EHRs. In general, COVID-19 clinical prediction models can broadly be categorized into risk, diagnosis, and prognosis models [20].

In Judson et al [21], a portal-based COVID-19 self-triage and self-scheduling tool was used to segment patients into 4 risk categories: emergent, urgent, nonurgent, and self-care, whereas the online telemedicine system in Liu et al [22] used LR to predict low-, moderate-, and high-risk patients by utilizing demographic information, clinical symptoms, blood tests, and computed tomography (CT) scan results.

In Schwab et al [3], various machine learning models were developed to predict patient outcomes from clinical, laboratory, and demographic features found in EHRs [23]. The authors reported that gradient boosting (XGB), random forests, and SVMs are the best-performing models for predicting COVID-19 test results, hospital admissions, and intensive care unit admissions for positive patients, respectively. A detailed list of clinical and laboratory features can be found in Wang et al [24], where the authors developed predictive models for the inpatient mortality in Wuhan using an ensemble of XGB models. Similarly, in Vaid et al [25], mortality and critical events for patients using XGB classifiers were predicted. Finally, a critical review on various diagnostic and prognostic models of COVID-19 used in clinical settings can be found in Wynants et al [20].

In Wagner et al [26], COVID-19 symptoms from unstructured clinical notes in the EHRs of patients subjected to COVID-19 polymerase chain reaction (PCR) testing were extracted. In addition, COVID-19 SignSym [27] was designed to automatically extract symptoms and related attributes from free text. Furthermore, the study by López-Úbeda et al [28] utilized radiological text reports from lung CT scans to diagnose COVID-19. Similar to our approach, López-Úbeda et al [28] first extracted concepts using a popular medical ontology [29] and then constructed a document representation using word embeddings [30] and concept vectors [28]. However, our methodology differs from theirs with respect to the extraction of relations between concepts, and moreover, our data set, comprising posts obtained from medical social media, is more challenging to work with, since social media posts exhibit greater heterogeneity in language than radiological text reports.

Finally, Sarker et al [4] published a COVID-19 symptom lexicon extracted from Twitter, which we compared our work to in the Discussion section.

## Methods

### Data

**Figure 2:**
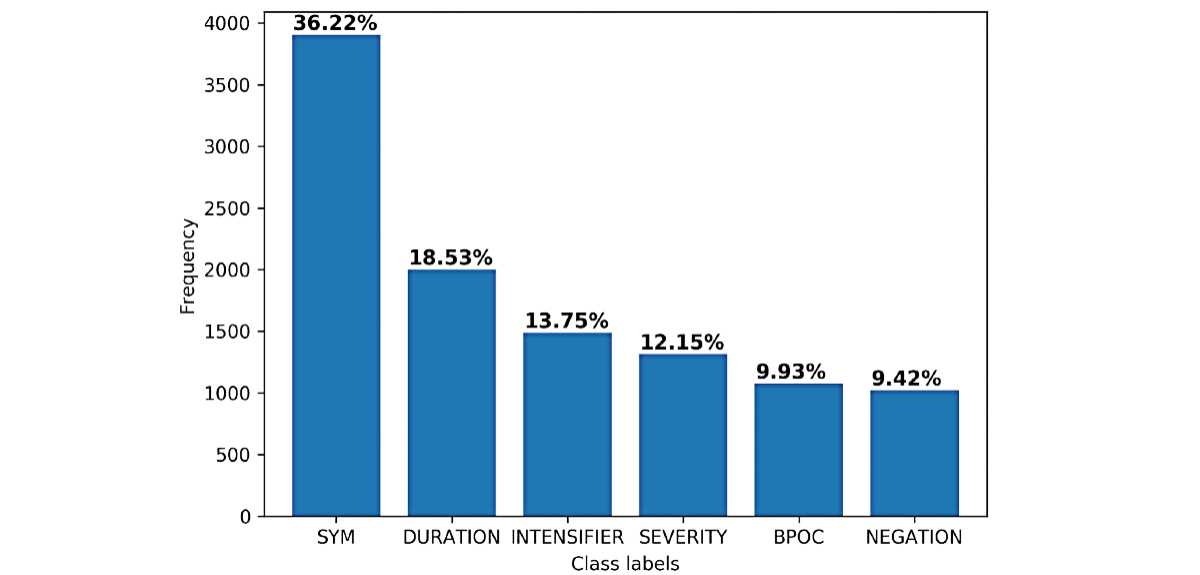
Frequency distribution of annotated classes/concepts from the text are shown. We have also shown the percentage of each class after discounting the OTHER labels. The average number of tokens per post was 130.17 (SD 97.83). BPOC: body part, organ, or organ component; SYM: symptoms.

We collected social media posts discussing COVID-19 medical conditions from a forum called *Patient* [31]. This a public forum that was created at the onset of the coronavirus outbreak in the United Kingdom. We obtained permission from the site administrator to scrape publicly available posts dated between April and June 2020. In addition, all user IDs and metadata were removed from the posts for the purpose of the study. After the posts were anonymized, and duplicates were removed, we randomly selected 500 distinct posts. The first author annotated these posts with the classes shown in Figure 2. The class labels represent symptoms and the related concepts: (1) duration; (2) intensifier, which increases the level of symptom severity; (3) severity; (4) negation, which denotes the presence or absence of the symptom or severity; and (5) affected body parts. We also annotated relations between a symptom and other concepts that exist at the sentence level. For example, the relation between a symptom and a severity concept is denoted as *(SYM, SEVERITY)*. The posts were then marked with concepts in different colors, and the relations were placed right after the symptom in square brackets, as shown in Figure 1. Each marked post was presented to the doctors using a web application, and they were asked 3 questions independently; see Figure 1. We called the doctors’ answers to questions 1 and 2 as the COVID-19 symptom triage and diagnosis, respectively. Thus, for each post, we had 3 independent answers from 3 doctors, which we denoted as A, B, and C, respectively; these corresponded to the last 3 authors of the paper and were assigned randomly.

#### Measurement of Agreement

To measure the agreement between the answers (recommendations and ratings) of the 3 doctors to questions 1 and 2 of Figure 1, we first calculated the proportion of observed agreement (*ρ*_o_), as suggested by de Vet et al [32], who stipulated that Cohen *κ* is actually a measure of reliability rather than agreement; we observed that *ρ*_o_ was high in all cases, as can be seen in Table 1. We noted that the paradoxical behavior of Cohen *κ* can arise when the absolute agreement (*ρ*_o_) is high [33]. This may occur when there is a substantial imbalance in the marginal totals of the answers, which we observed in the answers to question 1. Consequently, in addition to Cohen *κ*, we deployed a common solution to this problem, called the AC1 statistic devised by Gwet and coworkers [34,35].

We found that for question 1, the AC1 measure showed moderate agreement (in the middle of the moderate range) between A and B (0.55) and substantial agreement between A and C (0.72); see Landis and Koch [36] for the benchmark scale for the strength of agreement. For question 2, it turned out that said paradox did not occur, resulting in similar values for *κ* and AC1. The agreement between A and B (*κ*=0.64, AC1=0.67) and between B and C (*κ*=0.64, AC1=0.67) was substantial, while the agreement between A and C (*κ*=0.40, AC1=0.40) was on the boundary of fair and moderate; see Table 1.

It is important to note that COVID-19 is a novel virus disease, for which the doctors did not have prior experience or training before the first wave of the pandemic, and thus one would expect some difference of opinion. (We bear in mind that in our setting, the doctors can only see the posts and thus cannot interact with the patients as they would in a normal scenario.) Moreover, there are probable differences in risk tolerances between the doctors, which would lead to potentially different decisions and diagnoses.

**Table 1:** Pairwise agreement between pairs of doctors’ answers to questions 1 and 2; see Figure 1 for an example.

Pair	Question 1			Question 2		
	*ρ*_o_	*κ*	AC1	*ρ*_o_	*κ*	AC1
AB	0.65	0.26	0.55	0.73	0.64	0.67
BC	0.63	0.14	0.53	0.73	0.64	0.67
AC	0.77	0.28	0.72	0.51	0.40	0.40

### Problem Setting

#### Triage Classification for Question 1

We mapped the doctors’ recommendations from question 1 to ordinal values; the options *Stay at home*, *Send to a GP*, or *Send to a hospital* were transformed to the values 1, 2, and 3, respectively. To combine recommendations from 2 or more doctors, we first took their average. This result was rounded to an integer in 1 of 2 ways: either by taking the floor or by taking the ceiling. Considering the risk attitude prevalent among medical practitioners [11], we categorized the ceiling of the average to be *risk averse*, denoted by, for example, AB(R-a), and the floor to be *risk tolerant*, denoted by, for example, AB(R-t). Thus, for each patient’s post, we had in total 11 recommendations from 3 doctors for question 1. We constructed a hierarchical classification model for each of these recommendations, where the goal was to classify a post into 1 of the 3 options.

#### Diagnosis Classification for Question 2

To diagnose whether a patient has COVID-19 from their post, we first estimated the probability of having the disease by normalizing the rating (ie, given a rating, r, the probability of COVID-19, *P*r(COVID|r), which we termed the *ground-truth probability* (GTP), was simply *P*r(COVID|r) = (r – 1)/4.

Given our GTP estimates were discrete, we investigated 3 decision boundaries, denoted by LE, LT, and NEQ, based on a threshold value of 0.5 to classify a post as follows:

- LE: If Pr(COVID|r)≤0.5, then NO_COVID, else COVID.
- LT: If Pr(COVID|r)<0.5, then NO_COVID, else COVID.
- NEQ: If Pr(COVID|r)<0.5, then NO_COVID, elseif Pr(COVID|r)>0.5, then COVID.

Note that NEQ ignores cases on the 0.5 boundary.

### Methodology

**Figure 3:**
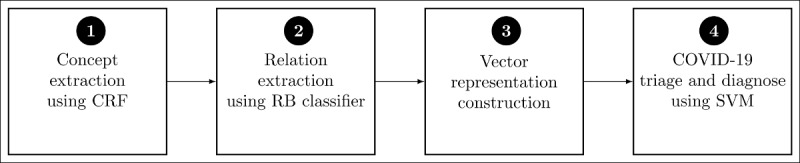
A block diagram of the COVID-19 triage-and-diagnosis text processing pipeline. CRF: conditional random field; RB: rule based; SVM: support vector machine.

A schematic of our methodology to triage and diagnose patients based on their social posts is shown in Figure 3. Here, the circles denote the steps followed in the pipeline. We now detail each of these steps.

#### Step 1: Concept Extraction

In the first step, we preprocessed each patient’s post by splitting it into sentences and tokens using General Architecture for Text Engineering (GATE) software’s (University of Sheffield) [37] built-in NLP pipeline. For each token in a sentence, we built discrete features that signal whether the token is a member of 1 of the following dictionaries: (1) Symptom, (2) Severity, (3) Duration, (4) Intensifier, and (5) Negation. The dictionaries were built by analyzing the posts while annotating them. We also utilized the MetaMap system [29], assuming that it contains all the necessary technical terms, to map tokens to 3 useful semantic categories: *Sign or Symptom*; *Disease or Syndrome*; and *Body Part, Organ, or Organ Component*. Due to the assumption regarding medical terms, the system does not expect any new additional terms, and thus we were justified in extracting concepts and relations in preprocessing steps. The preprocessed text was then used to build a concept extraction module to recognize the classes, shown in Figure 2, by applying a CRF [12]. A detailed description of our CRF training methodology can be found in Hasan et al [38]. The extracted concepts were then used for our next step to recognize the relations between concepts.

#### Step 2: Relation Extraction

The semantic relation between a symptom and other concepts, which we formally termed *modifiers*, was resolved using an unsupervised RB classifier algorithm. We first filtered all symptom and modifier pairs from a sentence within a predefined distance and then selected the closest modifier to a symptom to construct a relation. In total, we extracted 5 kinds of relations as follows: *(SYM, SEVERITY)*, *(SYM, DURATION)*, *(SYM, BPOC)*, *(SYM, NEGATION)*, and *(SYM, ?)*—here, SYM and BPOC refer to symptoms, and body part, organ, or organ component, respectively.

The severity modifiers were mapped to a scale of 1-5; the semantic meaning of the scale was *very mild*, *mild*, *moderate*, *severe*, and *very severe*, respectively. The duration modifiers were also mapped to real values in chunks of weeks. So, for example, *10 days* was mapped to the value *1.43*.

#### Step 3: Vector Representation

Fixed-length vector representations suitable as input for SVM classifiers were built as follows:

- *Symptom-only* vector representation: Let <S_0_, S_1_, . . . , S_n_> be a vector of symptoms constructed from the symptom vocabulary; for our data set, the number of unique symptom words/phrases was n=871. To construct the vector representation for a post, we extracted the concept, *SYM*, and the relation (*SYM*, *NEGATION*) and set S_i_ to 1, 0, or –1 according to whether the symptom was present, not present, or negated, respectively.
- *Symptom-modifier relation vector* representation: The symptom-modifier relation vector is a much larger vector than the symptom-only vector and comprises 3 appended vectors containing (1) the absence or presence of 110 unique body parts, (2) the absence or value of a symptom duration, and (3) the absence, negation, or value or a symptom severity.

#### Step 4: Triage and Diagnosis

We utilized SVM classification and regression models to triage and diagnose patients’ posts, respectively, from the vector representations described earlier. For question 1, the recommendation from a doctor or a combination of doctors was the class label of the post; see the Problem setting subsection in the Methods section for a description. To build a binary classifier, we first combined the *Send to a GP* and *Send to a hospital* recommendations to represent a single class, *Send*. The SVM was trained to distinguish between the *Stay at home* and the *Send* options; we called this *SVM classifier 1*. Next, the posts labeled as *Stay at home* were discarded and *SVM classifier 2* was built utilizing the remaining posts to classify the *Send to a GP* and *Send to a hospital* recommendations. This resulted in a hierarchical classifier for COVID-19 triage.

For diagnosing COVID-19 cases, we deployed a variant of the SVM, called *support vector regression* (SVR) [9], to estimate the probability of COVID-19. We used the GTP that was derived from answers to question 2 as the dependent variable. SVR takes as input a high-dimensional feature vector, such as a symptom-only or a symptom-modifier relation vector representation, as described earlier. Classification was performed using the 3 decision functions, LE, LT, and NEQ, described previously.

## Results

### Evaluation

**Figure 4:**
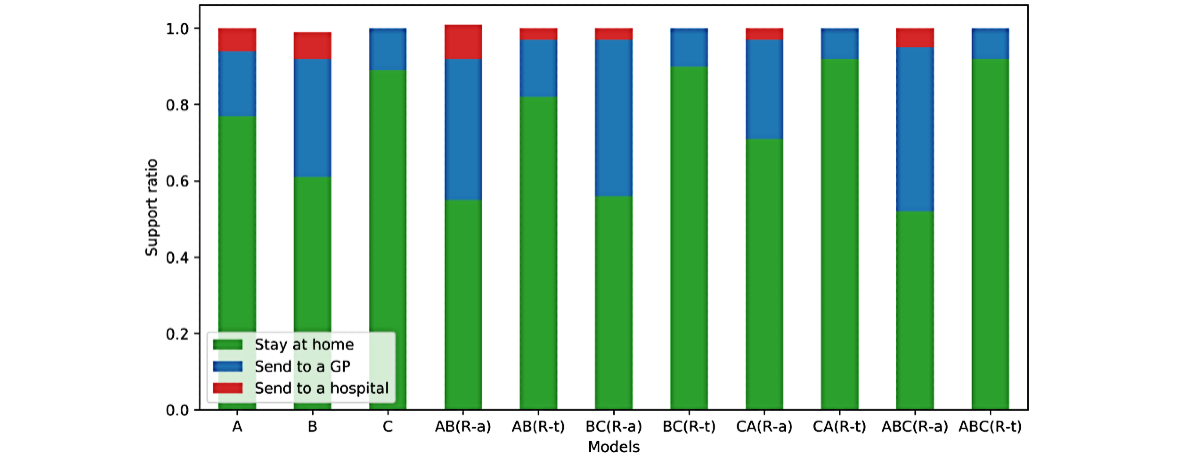
Support ratio of triage classes across models for question 1 classification tasks. Absolute numbers for the "Send to a hospital" class in test sets were as follows: A=10, B=12, AB(R-a)=14, AB(R-t)=5, BC(R-a)=6, AC(R-a)=5, and ABC(R-a)=9; the value for the remaining models was 0. GP: general physician.

We evaluated the performance of the CRF and SVM classification algorithms using the standard measures of precision, recall, and macro- and microaveraged F_1_ scores [39]. Macroaveraged scores were computed by considering the score independently for each class and then taking the average, while microaveraged scores were computed by considering all the classes together. As our data set was not balanced with *COVID* and *NO_COVID* classes, as can be seen in Figure 4, and we wished to give equal weight to all instances, we reported microaveraged scores for the SVR classification. In contrast, in the case of concept extraction, the *Other* class dominated. So, in this case, we reported the macroaveraged scores for the CRF classification results.

### Experimental Setup

For the CRF, we reported 3-fold cross-validated macroaveraged results. Specifically, we trained each fold by a Python wrapper [40] for CRFsuite; see Okazaki [41]. For relation extraction, we ran our unsupervised RB algorithm on the 500 posts and calculated the F_1_ scores by varying distances considering the 2 cases with and without stop words.

We constructed SVM binary classifiers, SVM classifier 1 and SVM classifier 2, using the Python wrapper for LIBSVM [42] implemented in Sklearn [43] with both linear and Gaussian *radial basis function* (RBF) kernels [10]. Similarly, SVR [44] was implemented using LIBSVM and was built with both linear and RBF kernels. The hyperparameters (C=10 for the penalty, γ=0.01 for the RBF kernel, and ε=0.5 for the threshold) were discovered using a grid search [43].

We simulated 2 cases for COVID-19 triage and diagnosis. First SVM and SVR models were trained with the ground truth to examine the predictive performance when they are deployed as stand-alone applications. Second, when trained with the predictions from the CRF and RB classifier, they resembled an end-to-end NLP application. To obtain a comparable result, the models were always tested with the ground truth. As a measure of performance, we reported macro- and microaveraged F_1_ scores for SVM classifiers and SVR, respectively.

### Evaluation Outcomes

**Figure 5:**
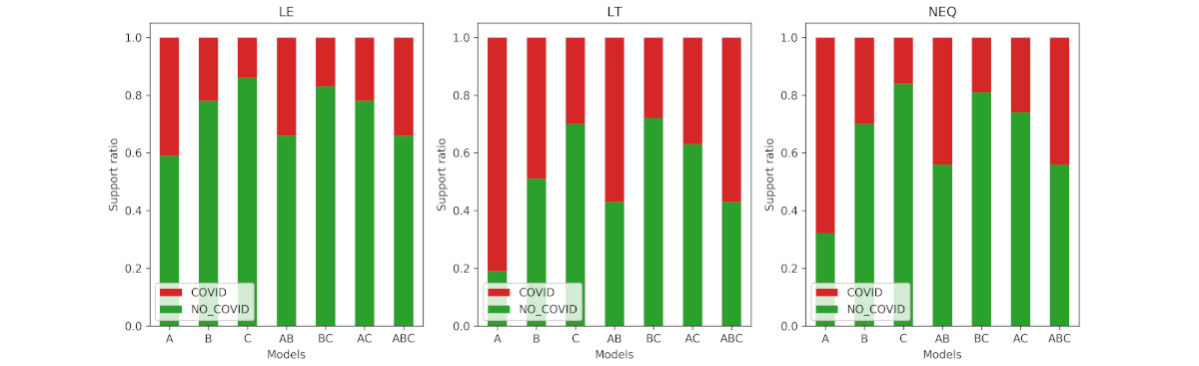
Support ratio of diagnosis classes across models and 3 decision functions for question 2 classification tasks.

The concept and relation extraction phases produced excellent and good predictive performances, respectively; see Tables 2 and 3. The triage classification results from question 1 are shown in Tables 4 and 5; the full enumeration can be seen in the first column. When we trained the models with the symptom-modifier vector representations from the ground truth, the results of SVM classifier 1 and SVM classifier 2 were in the range of 72%-93% and 83%-96%, respectively. The symptom-only vector representations produced results in the range of 71%-94% and 79%-95%. These results suggested that we can achieve good predictive performance for classifying *Stay at home* and *Send* and for *Send to a GP* and *Send to a hospital*. In general, risk-tolerant models achieved better performance than risk-averse models. However, since in the test set, posts with the label *Send to a hospital* were missing for some models (as can be seen from Figure 5), we could not report them. We reported macroaveraged F_1_ score results since question 1 was framed as a decision problem, where weights for the classes are a priori equal. The results obtained after training with CRF predictions were in similar ranges for both representations and classifiers. This is important, because it indicated that an end-to-end NLP application is likely to produce similar predictive performance.

Regarding question 2, when we trained the models with the symptom-modifier vector representation from the ground truth, the results of COVID-19 diagnosis were in the range of 72%-87%, 61%-76%, and 74%-87% for the LE, LT, and NEQ decision functions, respectively; see Table 6. The symptom-only vector representation produced results in the range of 70%-88%, 59%-79%, and 74%-87% for the LE, LT, and NEQ decision functions, respectively.

In general, NEQ models perform better due to the omission of borderline cases where the GTPs are exactly 0.5. The support ratios for each model for different decision functions are shown in Figure 4. When we trained the models with the symptom-modifier vector representation from the CRF predictions, the results were in the range of 68%-86%, 64%-76%, and 73%-87% for the LE, LT, and NEQ decision functions, respectively. This indicated that for diagnosis as well as triage, an end-to-end NLP application is likely to perform similarly to stand-alone applications. Here, we reported microaveraged F_1_ scores since, in our data set, *NO_COVID* cases dominated; this largely resembled the natural distribution in the population, where people who tested positive for coronavirus are a relatively low percentage in the whole population, even when the prevalence of the virus is high.

Finally, we trained our models using a linear kernel but found that the RBF dominates in most of the cases; however, linear kernels are useful in finding feature importance [45].

**Table 2:** Concept extraction using CRF^a^ on 3-fold cross-validation.

Label	Precision	Recall	F_1_ score	Support
SYM^b^	0.94	0.97	0.95	1300
SEVERITY	0.80	0.79	0.79	437
BPOC^c^	0.92	0.83	0.87	356
DURATION	0.87	0.91	0.89	667
INTENSIFIER	0.88	0.97	0.92	494
NEGATION	0.83	0.89	0.86	338
OTHER	0.99	0.98	0.98	16892
Macroaverage	0.89	0.89	0.89	—^d^

^a^CRF: conditional random field.

^b^SYM: symptoms.

^c^BPOC: body part, organ, or organ component.

^d^Not applicable.

**Table 3:** Relation extraction using RB^a^ classifier results on 3-fold cross-validation.

Distance	With stop words				Without stop words		
	Precision	Recall	F_1_ score	Precision		Recall	F_1_ score
2	0.74	0.63	0.68	0.74		0.64	0.69
3	0.75	0.67	0.71	0.75		0.67	0.71
4	0.75	0.69	0.72	0.75		0.69	0.72
5	0.75	0.71	0.73	0.74		0.71	0.73
6	0.74	0.72	0.73	0.74		0.72	0.73
7	0.73	0.73	0.73	0.73		0.73	0.73

^a^RB: rule based.

**Table 4:** Question 1: hierarchical classification results for the RBF^a^ kernel using the symptom-modifier relation vector.

Model			SVM^b^ classifier 1				SVM classifier 2				
		Precision		Recall	F_1_ score	Precision		Recall	F_1_ score		
**Trained on the ground truth**											
	A	0.82		0.91	0.86	0.73		0.95	0.83		
	B	0.73		0.77	0.75	0.81		0.99	0.89		
	C	0.85		0.98	0.91	—^c^		—	—		
	AB(R-a)	0.70		0.75	0.72	0.80		0.96	0.88		
	AB(R-t)	0.84		0.96	0.89	0.85		1.00	0.92		
	BC(R-a)	0.72		0.75	0.73	0.92		1.00	0.96		
	BC(R-t)	0.86		0.99	0.92	—		—	—		
	AC(R-a)	0.79		0.87	0.83	0.89		1.00	0.94		
	AC(R-t)	0.88		0.98	0.93	—		—	—		
	ABC(R-a)	0.70		0.76	0.73	0.89		0.99	0.93		
	ABC(R-t)	0.88		0.99	0.93	—		—	—		
**Trained on the CRF^d^ predictions**											
	A	0.81		0.89	0.85	0.72		0.91	0.80		
	B	0.74		0.74	0.74	0.81		0.99	0.89		
	C	0.85		0.96	0.90	—		—	—		
	AB(R-a)	0.73		0.71	0.71	0.81		0.96	0.88		
	AB(R-t)	0.84		0.94	0.88	0.84		1.00	0.92		
	BC(R-a)	0.74		0.71	0.72	0.92		1.00	0.96		
	BC(R-t)	0.88		0.98	0.93	—		—	—		
	AC(R-a)	0.81		0.85	0.83	0.89		1.00	0.94		
	AC(R-t)	0.88		0.98	0.93	—		—	—		
	ABC(R-a)	0.72		0.72	0.72	0.89		1.00	0.94		
	ABC(R-t)	0.89		0.98	0.93	—		—	—		

^a^RBF: radial basis function.

^b^SVM: support vector machine.

^c^Not applicable.

^d^CRF: conditional random field.

**Table 5:** Question 1: hierarchical classification results for the RBF^a^ kernel using the symptom-only vector.

Model		SVM^b^ classifier 1				SVM classifier 2				
		Precision	Recall	F_1_ score	Precision		Recall	F_1_ score		
**Trained on the ground truth**										
	A	0.83	0.91	0.87	0.74		0.85	0.79		
	B	0.71	0.81	0.76	0.81		0.98	0.89		
	C	0.87	0.97	0.92	—^c^		—	—		
	AB(R-a)	0.69	0.75	0.72	0.83		0.96	0.89		
	AB(R-t)	0.85	0.94	0.89	0.85		1.00	0.92		
	BC(R-a)	0.71	0.79	0.75	0.92		0.99	0.95		
	BC(R-t)	0.88	0.98	0.93	—		—	—		
	AC(R-a)	0.80	0.86	0.83	0.89		1.00	0.94		
	AC(R-t)	0.90	0.98	0.94	—		—	—		
	ABC(R-a)	0.68	0.74	0.71	0.90		1.00	0.95		
	ABC(R-t)	0.90	0.98	0.94	—		—	—		
**Trained on the CRF^d^ predictions**										
	A	0.84	0.89	0.87	0.74		0.82	0.78		
	B	0.74	0.79	0.77	0.82		0.98	0.89		
	C	0.86	0.95	0.90	—		—	—		
	AB(R-a)	0.72	0.76	0.73	0.83		0.92	0.87		
	AB(R-t)	0.87	0.93	0.90	0.84		0.98	0.90		
	BC(R-a)	0.72	0.78	0.75	0.92		0.99	0.95		
	BC(R-t)	0.87	0.97	0.92	—		—	—		
	AC(R-a)	0.80	0.86	0.83	0.89		1.00	0.94		
	AC(R-t)	0.89	0.95	0.92	—		—	—		
	ABC(R-a)	0.71	0.76	0.73	0.89		0.99	0.93		
	ABC(R-t)	0.90	0.95	0.92	—		—	—		

^a^RBF: radial basis function.

^b^SVM: support vector machine.

^c^Not applicable.

^d^CRF: conditional random field.

**Table 6:** Question 2: microaveraged F_1_ score results for different models and decision functions. Here, A, B, and C are 3 medical doctors (abbreviated as Dr) who took part in the experiment.

Model		Symptom-modifier vector			Symptom-only vector		
		LE	LT	NEQ	LE	LT	NEQ
**Trained on the ground truth**							
	A	0.72	0.61	0.78	0.70	0.59	0.74
	B	0.78	0.61	0.76	0.78	0.62	0.77
	C	0.87	0.75	0.87	0.88	0.75	0.87
	AB	0.72	0.66	0.74	0.74	0.65	0.75
	BC	0.84	0.76	0.84	0.85	0.79	0.86
	AC	0.81	0.73	0.81	0.83	0.74	0.83
	ABC	0.74	0.67	0.76	0.75	0.67	0.77
**Trained on the CRF^a^ predictions**							
	A	0.68	0.64	0.76	0.50	0.79	0.74
	B	0.76	0.64	0.77	0.78	0.57	0.74
	C	0.86	0.75	0.87	0.87	0.74	0.86
	AB	0.70	0.65	0.73	0.71	0.66	0.74
	BC	0.83	0.76	0.83	0.85	0.78	0.86
	AC	0.80	0.74	0.82	0.80	0.73	0.81
	ABC	0.72	0.69	0.76	0.74	0.69	0.77

^a^CRF: conditional random field.

## Discussion

### Principal Findings

This study demonstrates the potential to triage and diagnose COVID-19 patients from their social media posts. We presented a proof-of-concept system to predict a patient’s health state by building machine learning models from their narrative. The models were trained in 2 ways: using (1) ground-truth labels and (2) predictions obtained from the NLP pipeline. Trained models are always tested on ground-truth labels. We obtained good performances in both cases, which indicates that an automated NLP pipeline could be used to triage and diagnose patients from their narrative; see the Evaluation Outcomes subsection in the Results section. In general, health professionals and researchers could deploys triage models to determine the severity of COVID-19 cases in the population and diagnostic models to gauge the prevalence of the pandemic.

### Comparison With Prior Work

To quantify the important predictive features in the training set, we experimented with COVID-19 diagnosis using linear kernel SVR regression. More specifically, we used the symptom-only vector representation constructed from the ground truth. We summed feature weights for each S_i_ in <S_0_, S_1_, . . . , S_n_> from the 7 models and the 3 decision functions; see the Methods section. The features were then mapped to the categories found in the Twitter COVID-19 lexicon complied by Sarker et al [4]. The top 5 important features in our data set were *cough*, *anosmia/agusia*, *dyspnea*, *pyrexia*, and *fatigue*. Mizrahi et al [17] quoted 4 of these symptoms as the most prevalent coronavirus symptoms, strongly correlating with our findings.

**Figure 6:**
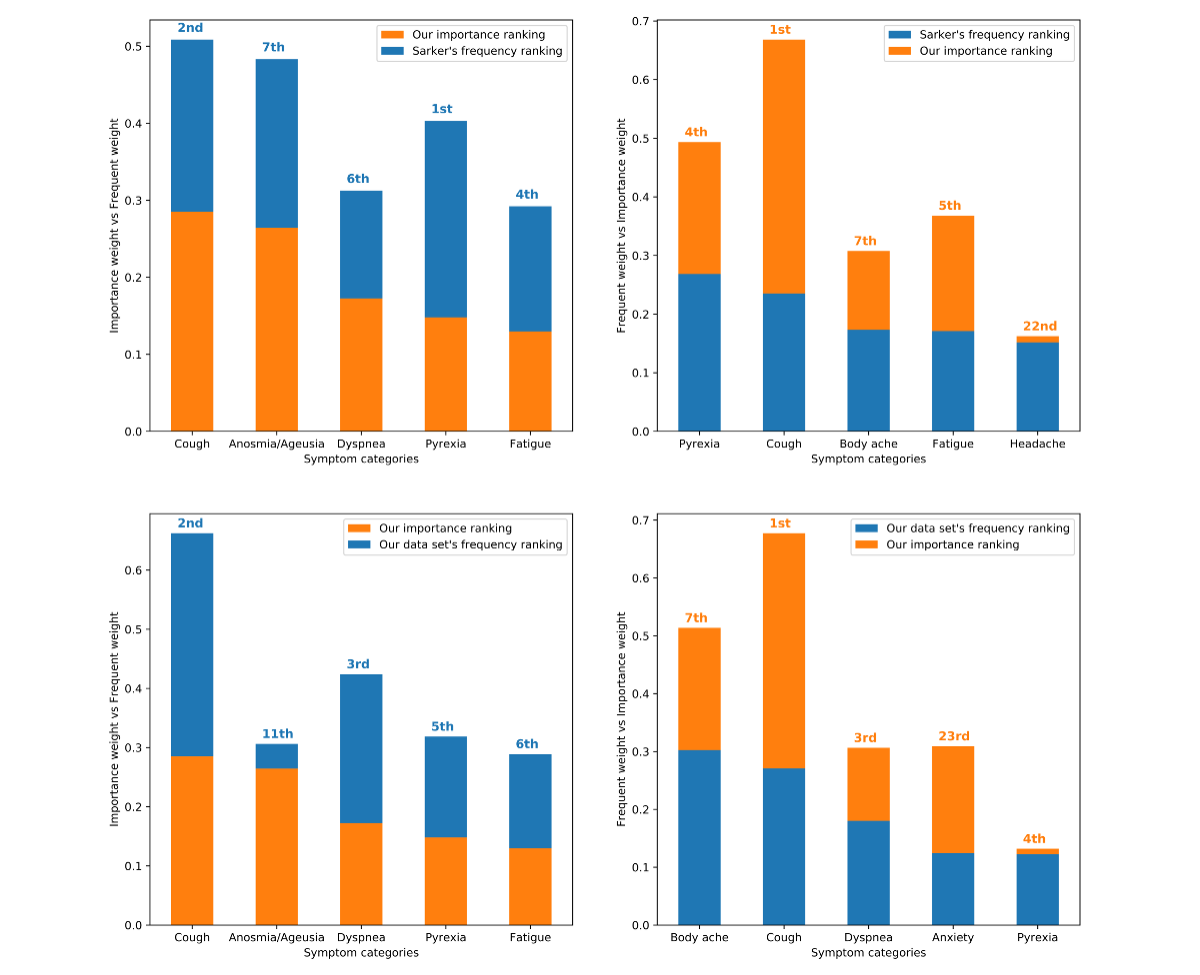
Feature comparison between our most important features and Sarker et al’s [4] most frequent symptoms (top row) and between our most important features and our most frequent symptoms (bottom row). The feature importance rankings are obtained from an SVM linear kernel using the symptom-only vector representation. SVM: support vector machine.

To compare our importance ranking with that of Sarker et al’s [4] frequent categories, we compiled the corresponding frequencies of our 5 most important symptoms. Normalized weights and frequencies were then plotted in Figure 6. The top-left stacked bar chart compares our 5 most important features with Sarker et al’s [4] frequencies. Cough was the most important symptom from our data set, where it was the second-most frequent. Anosmia/ageusia ranked second in our importance list, while it was seventh in the most frequent list. Pyrexia came first and fourth in both the frequent and importance lists, respectively.

The top-right chart in Figure 6 shows a comparison between Sarker et al’s [4] frequency ranking and our importance ranking. Here, we selected the top 5 most frequent symptoms from Sarker et al’s [4] frequency list and normalized them. These are *pyrexia*, *cough*, *body ache*, *fatigue*, and *headache*. We took the corresponding importance weights of these symptoms and plotted them in a stacked bar chart. Here, headache ranked 22^nd^ in our importance ranking, while it was 5^th^ in the frequency ranking. We found a large difference between the 2 rankings, implying that the top-most frequent symptoms are not necessarily the most important ones.

Next, we compared our most important feature weights with our data set’s frequency ranking using the methods described earlier. From the bottom-left stacked bar chart of Figure 6, we observed that anosmia/ageusia were relatively low in order in the frequency ranking (ie, 11^th^). As in Sarker et al’s [4] ranking, cough came second in our data set’s frequency ranking.

Finally, the bottom-right chart in Figure 6 refers to the comparison between our data set’s frequency and importance rankings for the corresponding symptoms. We observed that anxiety ranked 4^th^ in the frequency list, while it was low (ie, 23^rd^) in the importance ranking.

### Limitations

It is worth reiterating that social media posts, which are known to be noisy, are not on a par with the consultation that a patient would have with a doctor. We stress that the aim of this study is to extract useful information at a population level, rather than to provide an actionable decision for an individual via social media posts. Our manually annotated data set has 2 main limitations. First, having only 3 experts limited the quality of our labeling, although we deem this study to be a proof of concept. A larger number of experts, including more senior doctors, would be beneficial in a follow-up study. The robustness of our results could be further improved by both increasing the size of our data set and introducing posts from several alternate sources. Given that the posts come from social media, it is not clear whether the results could be used as such in a diagnostic system, without combining them with actual consultations. However, it is worth noting that medical social media, such as the posts we used herein, may uncover novel information regarding COVID-19.

### Conclusion

The coronavirus pandemic has drawn a spotlight on the need to develop automated processes to provide additional information to researchers, health professionals, and decision makers. Medical social media comprises a rich resource of timely information that could fit this purpose. We have demonstrated that it is possible to take an approach that aims at the detection of COVID-19 using an automated triage and diagnosis system in order to augment public health surveillance systems, despite the heterogeneous nature of typical social media posts. The outputs from such an approach could be used to indicate the severity and estimate the prevalence of the disease in the population.

## Abbreviations

BPOC: body part, organ, or organ component
CRF: conditional random field
CT: computed tomography
EHR: electronic health record
GP: general physician
GTP: ground-truth probability
LR: logistic regression
NLP: natural language processing
RB: rule based
RBF: radial basis function
SVM: support vector machine
SVR: support vector regression
SYM: symptoms
XGB: gradient boosting
